# Genetic and environmental variation in condition, cutaneous immunity, and haematocrit in house wrens

**DOI:** 10.1186/s12862-014-0242-8

**Published:** 2014-12-04

**Authors:** Scott K Sakaluk, Alastair J Wilson, E Keith Bowers, L Scott Johnson, Brian S Masters, Bonnie GP Johnson, Laura A Vogel, Anna M Forsman, Charles F Thompson

**Affiliations:** Behavior, Ecology, Evolution and Systematics Section, School of Biological Sciences, Illinois State University, Normal, IL USA; Centre for Ecology and Conservation, School of Biosciences, University of Exeter, Penryn, UK; Department of Biology, Towson University, Towson, MD USA; Now at Department of Ecology and Evolutionary Biology, Cornell University, Ithaca, NY USA

**Keywords:** Animal model, Condition, Haematocrit, Heritability, Genetic variation, Immunity, Life-history theory, PHA, *Troglodytes aedon*

## Abstract

**Background:**

Life-history studies of wild bird populations often focus on the relationship between an individual’s condition and its capacity to mount an immune response, as measured by a commonly-employed assay of cutaneous immunity, the PHA skin test. In addition, haematocrit, the packed cell volume in relation to total blood volume, is often measured as an indicator of physiological performance. A multi-year study of a wild population of house wrens has recently revealed that those exhibiting the highest condition and strongest PHA responses as nestlings are most likely to be recruited to the breeding population and to breed through two years of age; in contrast, intermediate haematocrit values result in the highest recruitment to the population. Selection theory would predict, therefore, that most of the underlying genetic variation in these traits should be exhausted resulting in low heritability, although such traits may also exhibit low heritability because of increased residual variance. Here, we examine the genetic and environmental variation in condition, cutaneous immunity, and haematocrit using an animal model based on a pedigree of approximately 2,800 house wrens.

**Results:**

Environmental effects played a paramount role in shaping the expression of the fitness-related traits measured in this wild population, but two of them, condition and haematocrit, retained significant heritable variation. Condition was also positively correlated with both the PHA response and haematocrit, but in the absence of any significant genetic correlations, it appears that this covariance arises through parallel effects of the environment acting on this suite of traits.

**Conclusions:**

The maintenance of genetic variation in different measures of condition appears to be a pervasive feature of wild bird populations, in contradiction of conventional selection theory. A major challenge in future studies will be to explain how such variation persists in the face of the directional selection acting on condition in house wrens and other species.

## Background

An animal’s condition is defined by the pool of resources available to allocate to the production and maintenance of traits that enhance fitness, and reflects an individual’s ability to acquire food, avoid predators, and resist disease [1]. Body condition is critically important to life history because a variety of traits affecting fitness are often condition dependent, such as those involved in mate attraction [1] or disease resistance [2]. A number of morphological and physiological indices for measuring condition have been devised, although the interpretation of these measures is often controversial and their validity frequently questioned [3]. In field studies of birds and mammals, an individual’s condition is often measured as body mass corrected for structural body size, which is often derived from the residuals of a regression of mass on body size [4]. Condition measured in this manner is assumed to reflect lipid reserves [5], a major form of energy storage in birds that is used to fuel the various processes that promote individual fitness (i.e., flight, migration, reproduction). Indeed, body-mass based measures appear to be tightly linked with fitness in a number of vertebrate species [6].

In studies of wild bird populations, the condition of both nestlings and breeding adults is often measured in the context of understanding life-history variation [7]. For example, all else being equal, individuals in better condition should be able to invest more in resource-limited traits associated with survival and/or reproduction. Such traits include an individual’s capacity to mount an immune response, which is an important component of maintenance [8-11]. This is particularly relevant to nestlings that, owing to their lack of mobility, are especially vulnerable to parasites and pathogens. A standard method employed for measuring cutaneous immune activity in wild birds is the phytohaemagglutinin (PHA) skin test, an assay that involves injecting a novel plant mitogen, PHA, into the wing prepatagium of the bird and measuring the ensuing swelling 24 h later. The swelling is taken as a measure of cutaneous immune responsiveness, which includes both innate and adaptive components of the immune system [12-15]. Although the conventional expectation has been that birds in good condition should be capable of mounting more robust immune responses [7], this expectation has been met in some studies [9,16], but not in others [11,17].

In addition to morphological measures of condition, haematocrit, the packed volume of red blood cells in relation to total blood volume, is often measured as a presumed physiological indicator of condition in field studies of wild birds [18,19]. Because haematocrit is directly related to oxygen uptake, it is typically regarded as a measure of physiological performance [18-20], but its value as an indicator of condition or health state remains uncertain [11,17,21]. A growing body of evidence suggests, however, that physiological variation in haematocrit at different life-history stages is tied directly to such fundamental processes as reproduction, migration, and the acquisition of flight [22], and so there is reason to believe that haematocrit may be linked, at least under some circumstances, to fitness.

Over the past 10 years, we have routinely measured condition, PHA response, and haematocrit in a wild population of house wrens, *Troglodytes aedon*, in north-central Illinois, USA [23-26]. A multi-year study of a large subset of the nestlings measured over this time period revealed that those in the best condition and with the strongest PHA responses were more likely to be recruited to the breeding population and more likely to breed through two years of age [27]. The relationship between haematocrit and fitness was somewhat more complex, as intermediate haematocrit values resulted in the highest recruitment to our population, suggestive of stabilizing selection [27].

If, as our data on recruitment and subsequent breeding success would suggest, condition, immune response to PHA injection, and haematocrit in this population are subject to strong natural selection, we would anticipate that most of the underlying genetic variation in these traits would be exhausted [28]. However, a number of studies of wild bird populations have reported significant heritable variation in each of these traits [condition: [6,29,30]; PHA response: [10,31,32]; haematocrit: [33]]. This is, perhaps, most surprising in the case of condition, which is expected to reflect an individual’s current nutritional state and thus be determined primarily by environmental variation [6]. Here, we examine the genetic and environmental variation in condition, cutaneous immune responsiveness, and haematocrit over a three-year period (2004–2006) during which we used DNA profiling to determine the parentage of nestlings. Specifically, our objectives were to determine the degree of heritable variation in each of these traits and to measure the phenotypic and genetic correlations among these characters.

## Methods

### Study animals

House wrens are small (10–12 g), insectivorous songbirds that are sexually monomorphic in size and plumage. As obligate cavity-nesters, house wrens readily accept nestboxes within which to build their nests. Upon arrival at the study area in north-central Illinois after spring migration, females select a male that is defending a nest site and, after completing the nest, lay a clutch of 4–8 eggs. About half of the females that successfully rear their first brood produce a second [26,34,35]. Only female house wrens incubate the eggs and brood the nestlings, but both adults provision nestlings and fledglings. House wrens are usually socially monogamous, but social polygyny occurs and extra-pair fertilizations are common in the study population [23,36]. Additional information concerning the breeding biology of house wrens is provided by Johnson [37].

We conducted this study during the 2004–2006 breeding seasons on the Mackinaw study site (40°40′N, 88°53′W) in a second-growth, deciduous forest bordering the Mackinaw River. Nestboxes (*N* =700) of uniform construction [38] were spaced 30 m apart along north–south transects separated by 60 m (Figure one in [39]). All nestboxes were mounted on 1.5-m metal poles that had been greased or under which 48.3-cm diameter aluminium baffles had been mounted to discourage nest predators. The nestboxes used in this study (*N* =301) were located in three semi-isolated neighbourhoods within the study area [23]. All activities complied with the Illinois State University Institutional Animal Care and Use Committee (Protocol No.17-2003) and United States Geological Survey banding permit 09211.

### Field procedures

We visited all nestboxes every 1–3 days, noting their contents, the behaviours of any wrens present, and male identity as revealed by unique combinations of coloured, Darvic leg rings. We trapped and individually ringed any previously uncaught females when incubating or males when provisioning nestlings. To obtain DNA for paternity analyses, we collected a blood sample from adults upon capture and from all nestlings. On brood-day 11 or 12 (brood-day 0 is the day the first egg of a clutch hatches), we ringed nestlings with a numbered aluminium band, weighed them on an electronic balance (Acculab, Pocket Pro 250-B) to the nearest 0.1 g, and measured their right tarsus length with dial callipers to the nearest 0.1 mm. At the same time, we obtained a blood sample (≈75 μL) from the brachial vein in heparinized microcapillary tubes that were stored on ice in coolers in the field. Blood was taken to the laboratory later the same day to be centrifuged at 1,610 g for 60 s (Hematastat II, Separation Technologies) to separate cellular and plasma components. We measured haematocrit as the percentage of whole blood constituted by red blood cells, using the mean of three measurements. Red blood cells were stored in lysis buffer at 4°C and plasma at −20°C until further analysis.

We induced a cutaneous immune response in nestlings on brood-day 11 by injecting 50 μL of sterile phosphate buffered saline (PBS) containing phytohaemagglutinin (Sigma Aldrich, St. Louis, MO, USA) at a concentration of 5 mg mL^−1^ [40] into their prepatagium (wing-web). Phytohaemagglutinin (PHA), a plant-derived mitogen, induces a measurable tissue swelling as a result of responses from both the innate and adaptive axes of the immune system [12]; but see [15]. We used the change in wing-web thickness (mean of three successive readings before and 24 h after PHA injection) as a measure of cutaneous immune activity. Wing-web thickness was measured with a Mitutoyo thickness gauge (no. 547–500, Mitutoyo America Corp., Aurora, IL, USA). The repeatability of haematocrit and the PHA response was 0.99 and 0.95, respectively [41].

### Determination of parentage

We isolated DNA from blood samples using a high-salt extraction protocol following Bruford et al. [42]. Polymerase chain reaction (PCR) amplifications were carried out in 15-μL volumes containing 200 μM dNTPs, 2.5 mM MgCl_2_, 1X PCR Buffer II (Applied Biosystems), and 0.133 μM forward and reverse primers. We used a thermal profile that followed the touchdown protocol described in Johnson et al. [43]. Forward primers were fluorescently labelled, and PCR products were analysed using a Beckman Coulter CEQ 8000 Genetic Analysis System. We typed all samples at three loci: TA-C3 (B)2 [44], Mcyμ4 [45], and LTMR6 [46]. When more resolution was needed, two additional loci, TA-A5-15 and TA-B4-2 [44], were used. We analysed allele data using Cervus 2.0. No locus deviated significantly from Hardy-Weinberg equilibrium, with the exception of TA-A5-15, whose null allele frequency was estimated at 0.094.

Attendant females matched nestlings at all loci with rare exceptions (i.e., six cases in which there was a mismatch at a single locus, attributable to mutation) and were assumed to be the genetic parent in all cases. At some nests, we were unable to obtain blood samples from attending adults, in which case, maternity of the attending female was assumed because intraspecific brood parasitism does not occur in our population [36]; paternity of nestlings in these cases was scored as unknown. Nestlings that matched attendant males at all loci were assigned within-pair paternity, and those that failed to match attendant males at two or more loci were designated as extra-pair. A few nestlings failed to match attendant males at one locus, so we re-typed at the anomalous locus to prevent typing error. If they still failed to match the attendant male, we typed them at additional loci to attempt to resolve the anomaly. In some cases, they failed to match the attendant male at one or more of these additional loci and were therefore designated as extra-pair nestlings. If the probability of false assignment [47] based on matching loci was lower than 0.005, then nestlings were designated as within-pair nestlings. In some cases, the paternity of nestlings could not be resolved as a result of loss of sample or when repeated attempts to genotype failed to produce reliable allele data for one or more loci because of the poor quality of the DNA sample. Exclusion probabilities for each locus were Mcyμ4, 0.811; LTMR6, 0.691; TA-C3(B)2, 0.841; TA-B4-2, 0.641; TA-A5-15, 0.464. For the three-locus set and five-locus set exclusion, probabilities were 0.991 and 0.998, respectively. Overall, the probability of false assignment for nestlings designated as within-pair was <0.008.

**Figure 1:**
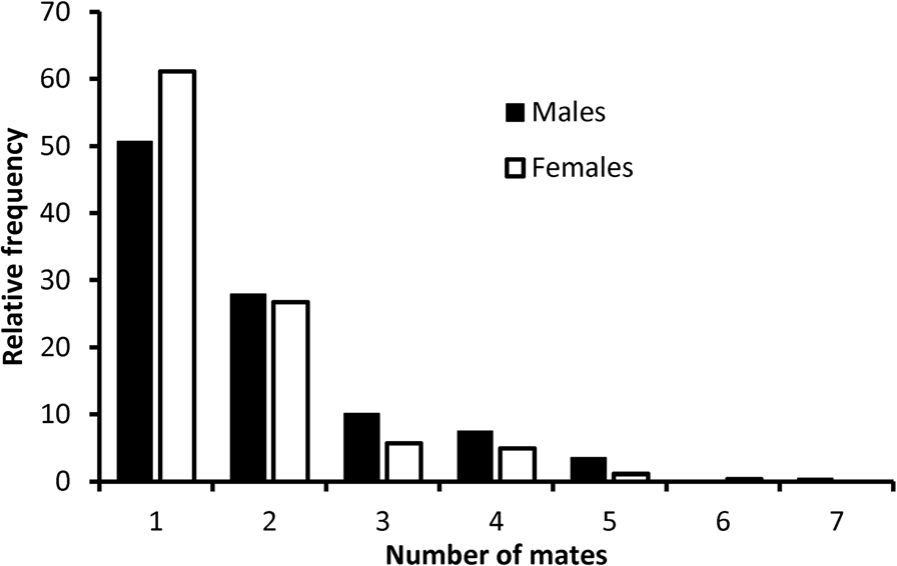
**Relative frequency of male house wrens males siring young with different numbers of females (solid bars) and the relative frequency of female house wrens producing young with different numbers of males (open bars).**

Based on these methods, we were able to construct a pedigree comprised of 2,768 house wrens, in which we were able to identify both parents of 1,661 individuals and only the mother of an additional 552 individuals. The pedigree spanned three generations, and included 229 paternal and 265 maternal identities. Not all phenotypic traits were measured on all individuals; sample sizes and mean values (±SE) for all traits are shown in Table 1. Unlike cross-fostering studies, our ability to separate nest effects (i.e., the effect of common environment) from genetic effects depends on the extent of extra-pair paternity and mate-switching across different reproductive episodes. As noted earlier, extra-pair paternity is common in our house-wren population (35-40% of broods; [23,36]), and, on our study area, nearly 60% of pairs switch mates across broods produced during the same breeding season [34]. Birds returning to the study area in subsequent years (as was the case with many of the birds in our pedigree) invariably pair with a different mate from the one with which they have paired the previous year (pers. obs.). As a consequence of the collective force of these factors, a high proportion of females in our pedigree produced young with more than one male (39%), and an even higher proportion of males sired young with more than one female (49%) (Figure 1). Thus, our pedigree is well suited to separating common nest effects and genetic effects.

**Table 1:** **Mean trait values (and associated sample sizes) for nestling house wrens included in the animal model analysis**

**Trait**	***N***	**Mean**	**SD**
Mass (g)	2199	9.68	0.89
Tarsus (mm)	1299	18.48	0.69
PHA (mm)	1945	0.60	0.32
Haematocrit (%)	1952	41.46	6.49

### Determination of sex

Sex was determined by amplifying sex-specific introns of the CHD-1 gene. Polymerase chain reactions (PCR) were carried out with sexing primers 1237L and 1272H [48] using a touchdown protocol as described in Johnson et al. [43], and the products electrophoresed through 2% agarose gels and stained with ethidium bromide. DNA isolated from adult house wrens of known sex was included in all sets of PCR runs as controls, and their PCR products were always electrophoresed with those of nestling samples. Because some nestlings did not survive to brood-day 11–12 and some DNA samples did not successfully amplify, not all nestlings in each nest were sexed.

### Genetic analyses

Data were analysed using animal models fitted in ASReml (version 3). An animal model is a form of linear mixed-effect model in which an individual’s genetic merit is included as a random effect allowing estimation of the additive genetic (co)variance provided pedigree data are available [49,50]. We first fitted univariate models to estimate the additive variance (V_A_) for each trait (tarsus, PHA, haematocrit, mass). Nest identity was included as an additional random effect to minimise the potential for upward bias in genetic variance from common environment effects. Nest effects are assumed to be normally distributed with a mean of zero and a variance to be estimated (V_nest_). Similarly, we assume that residuals from the model are uncorrelated and normally distributed, with the residual variance (V_R_) arising from unmodelled environmental effects and/or measurement error. Fixed effects included year (2004, 2005, 2006), and sex (unknown, male, female) as three-level factors for all traits. In addition, we included a cubic regression of tarsus length as a fixed effect in the model of mass. By correcting for the average mass-tarsus length relationship, variance components (and derived parameters) from our model of mass can be viewed as pertaining to body condition (i.e. mass “corrected” for skeletal size). For each trait we determined heritability (h^2^) as the ratio of V_A_ to V_P_ (with V_P_ estimated as the sum of the variance components; i.e., conditional on fixed effects) and the corresponding proportion of variance explained by nest effects (denoted n^2^). To test the genetic basis of hypothesised relationships among traits, we then fitted a multivariate model of all traits simultaneously with fixed effects as described above but no random effects. Note that the cubic function of tarsus length was included as a fixed effect on mass only, not the other response variables. This yielded an estimate of the phenotypic variance-covariance matrix (conditional on fixed effects) that we denote **P**. We then added individual genetic merit and nest effects back into the model to partition **P** into additive genetic (**G**), nest (**N**) and residual (**R**) matrices. Statistical inference was based on likelihood-ratio tests (LRT) for random effects and Wald F-statistics for fixed effects. For LRT of single random-effect terms in the univariate models, we assume the test statistic to be asymptotically distributed as a 50:50 mix of χ^2^_0_ and χ^2^_1_ [51]. For comparing multivariate models with differing random effects, we (conservatively) set the degrees of freedom to equal the number of additional (co)variance parameters estimated in the more complex model.

## Results

Fixed-effect estimates for nestling house wren traits are shown in Table 2 and their associated heritabilities are provided in Table 3. Nest effects accounted for a major portion of the variation in all traits, ranging from 21% of the variation in tarsus length to 54% of the variation in the PHA response (Table 3). Notwithstanding this large amount of environmental variation, both nestling haematocrit and condition (i.e., size-adjusted mass) were significantly heritable, with additive genetic variance accounting for about 15% of the phenotypic variation in each case (Table 3). Nestling condition differed across years, and male nestlings were of significantly higher body condition than female nestlings (Table 2). There were no significant year effects or sex differences in nestling haematocrit (Table 2).

**Table 2:** **Univariate models showing fixed effect estimates for nestling house wren traits**

**Trait/Effect**	**Level**	**Coefficient ± SE**	**DF**_**num**_	**DF**_**den**_	***F***	***P***
TARSUS						
μ		18.33 ± 0.094	1	177.5	83199	<0.001
Year^1^	2005	−0.066 ± 0.079	2	233.2	2.41	0.093
	2006	0.0728 ± 0.075				
Sex	Male	0.275 ± 0.074	2	637.5	22.83	<0.001
	Female	0.031 ± 0.075				
HAEMATOCRIT						
μ		42.06 ± 0.98	1	319.5	5003	<0.001
Year^1^	2005	−1.33 ± 0.87	2	394.3	1.18	0.310
	2006	−1.01 ± 0.87				
Sex	Male	0.517 ± 0.636	2	1308	0.59	0.550
	Female	0.311 ± 0.635				
PHA						
μ		0.785 ± 0.040	1	320.1	491.5	<0.001
Year^1^	2005	−0.327 ± 0.035	2	386.6	49.95	<0.001
	2006	−0.106 ± 0.032				
Sex	Male	−0.047 ± 0.031	2	1340	1.32	0.270
	Female	−0.039 ± 0.031				
MASS (“Condition”)						
μ		9.81 ± 0.105	1	316.9	17361	<0.001
Year^1^	2005	−0.309 ± 0.088	2	431.8	8.46	<0.001
	2006	−0.346 ± 0.088				
Sex	Male	0.225 + 0.079	2	1505	5.53	0.004
	Female	0.157 + 0.079				
Tarsus		0.519 + 0.034	1	2101	235.7	<0.001
Tarsus^2^		−0.149 + 0.027	1	2100	31.37	<0.001
Tarsus^3^		−0.027 + 0.004	1	2035	54.2	<0.001

^1^Year effects for 2005 and 2006 are shown relative to the predicted mean in 2004.

**Table 3:** **Estimated variance components of nestling house wren traits (conditioned on fixed effects) under the animal model, heritabilities (h** ^**2**^ **), and nest effects (n** ^**2**^ **)**

**Trait/Component**	**Estimate ± SE**	**Full model LnL**	**Reduced LnL**	**χ** ^**2**^	***P***
TARSUS					
V_P_	0.466 ± 0.021				
V_A_	0.052 ± 0.057	−102.58	−102.99	0.83	0.180
V_nest_	0.096 ± 0.026	−102.58	−111.62	18.08	<0.001
V_residual_	0.318 ± 0.038				
h^2^	0.113 ± 0.121				
n^2^	0.206 ± 0.054				
HAEMATOCRIT					
V_P_	42.47 ± 2.01				
V_A_	6.34 ± 3.26	−4253	−4256	4.86	0.0137
V_nest_	20.18 ± 2.25	−4253	−4374		<0.001
V_residual_	15.95 ± 2.00				
h^2^	0.149 ± 0.076				
n^2^	0.475 ± 0.039				
PHA					
V_P_	0.0883 ± 0.0044				
V_A_	0.0068 ± 0.0065	1811	1810	1.24	0.133
V_nest_	0.0477 ± 0.0049	1811	1658	305.9	<0.001
V_residual_	0.0338 ± 0.0040				
h^2^	0.077 ± 0.073				
n^2^	0.540 ± 0.038				
MASS (“Condition”)					
V_P_	0.712 + 0.031				
V_A_	0.096 + 0.055	−373.8	−375.4	3.166	0.038
V_nest_	0.315 + 0.035	−373.8	−478.3	209.0	<0.001
V_residual_	0.300 + 0.034				
h^2^	0.135 + 0.077				
n^2^	0.443 + 0.039				

Neither tarsus length nor PHA response were significantly heritable (Table 3). There was a significant difference between male and female nestlings in tarsus length, with males exhibiting longer tarsi (Table 2). Tarsus length did not vary across the three years of the study. There was no sex difference in the PHA response, but PHA response varied across the three years of the study (Table 2).

Our multivariate modelling with no random effects included showed that **P** contained significant among-trait covariance (LRT comparison of model with full **P** matrix to one with diagonal elements only, i.e., all covariance terms set to zero; χ^2^_6_ = 136, P < 0.001). This result was driven by significant positive phenotypic correlations between nestling body condition and each of the other three traits, tarsus length, PHA response, and haematocrit (Table 4). There was also a small, but significant correlation between tarsus length and PHA response. Partitioning the **P** matrix provided support for statistically significant covariance in **N** (full covariance matrix (full) versus variance only matrix (diagonal) LRT; χ^2^_6_ = 22.4, P = 0.001) and **R (**full versus diagonal matrix LRT; χ^2^_6_ = 38.5, P < 0.001). Among broods (i.e., nests), nestling body condition was positively associated with both tarsus length and haematocrit, but was not correlated with PHA response (Table 4). However, there was no evidence of significant covariance structure in **G (**full versus diagonal matrix LRT; χ^2^_6_ = 3.24, P = 0.778), and pairwise genetic correlations were characterised by large standard errors in all cases (Table 4).

**Table 4:** **Phenotypic covariance structure (P) and its additive genetic (G), nest (N) and residual (R) components**

**Matrix**	**Trait**	**Tarsus**	**PHA**	**Hematocrit**	**Condition**
**P**	Tarsus		**0.053 (0.026)**	0.021 (0.023)	**0.605 (0.020)**
	PHA	**0.011 (0.005)**		−0.004 (0.025)	**0.108 (0.023)**
	Haematocrit	0.093 (0.125)	−0.007 (0.049)		**0.138 (0.022)**
	Condition	**0.382 (0.02)**	**0.03 (0.007)**	**0.835 (0.139)**	
**G**	Tarsus		−0.130 (0.759)	−0.648 (0.701)	0.516 (0.434)
	PHA	−0.002 (0.0140)		0.020 (0.545)	0.361 (0.529)
	Haematocrit	−0.334 (0.303)	0.004 (0.095)		−0.384 (0.470)
	Condition	0.038 (0.045)	0.01 (0.015)	−0.306 (0.329)	
**N**	Tarsus		0.082 (0.114)	0.077 (0.122)	**0.436 (0.105)**
	PHA	0.006 (0.008)		−0.035 (0.080)	0.097 (0.080)
	Haematocrit	0.115 (0.183)	−0.035 (0.08)		**0.278 (0.077)**
	Condition	**0.083 (0.026)**	0.012 (0.01)	**0.729 (0.217)**	
**R**	Tarsus		0.074 (0.082)	0.124 (0.088)	**0.629 (0.057)**
	PHA	0.008 (0.008)		0.050 (0.087)	0.066 (0.079)
	Haematocrit	0.283 (0.199)	0.037 (0.066)		**0.175 (0.080)**
	Condition	**0.221 (0.03)**	0.008 (0.009)	**0.442 (0.207)**	

**P** was estimated from a multivariate model with no random effects while individual identification and nest were then included to decompose the covariance matrix into **G**, **N** and **R**. Values below the diagonals are covariances (with SE) and values above the diagonals are the corresponding correlations. Bold values denote nominally significant parameter at *P* <0.05 based |estimate| ≥2SE.

## Discussion

In keeping with a number of other studies of wild bird populations, the majority of variation in condition, cutaneous immune response, and haematocrit in our house wren population could be accounted for by nest effects, attesting to the paramount role of the early environment in influencing the expression of these traits. This is, perhaps, not surprising given that these traits often are subject to strong natural selection, which might be expected to exhaust any genetic variation underlying these traits [28,52-54]. Nevertheless, two of the traits measured in this study, condition and haematocrit, were significantly heritable, while our measure of cutaneous immune responsiveness was not, in seeming contrast to some studies showing heritable variation in the PHA response [10,31,32], and other cross-fostering studies showing an absence of nest-of-origin effects on nestling haematocrit [17,21]. Below, we explore the proximate underpinnings of the environmental and genetic factors influencing these important fitness-related traits, and the phenotypic and genetic relationships among them.

Nest effects accounted for the largest portion of phenotypic variation in both tarsus length and condition, but unlike tarsus length, condition was significantly heritable. Although we can only speculate as to the sources of environmental variation for these two traits, likely candidates include differential provisioning among broods [39,55] or spatial variation in the availability of insect prey [16]. Parasites might also contribute to the observed nest effects on these traits [33], as nestlings in our house wren population are frequently infested with ectoparasitic mites [56]; however, in an earlier study of our population, nestling mass was not affected by ectoparasite numbers [56] and in a different house wren population, ectoparasites had no detectable effect on nestling tarsus and only a slight effect on mass [57]. There was also significant variation among years in nestling condition, which might also reflect temporal variation in prey availability arising from annual variation in rainfall and summer temperatures.

The absence of significant genetic variation in tarsus length was surprising, given that the heritability of tarsus length in birds typically hovers around 0.5 [58]. However, Christe et al., [33] also found that tarsus length was not significantly heritable in house martins, *Delichon urbicum*. Because environmental factors such as parasites and food availability can profoundly influence variation in tarsus length, heritability of tarsus may vary according to the environment in which it is measured. Moreover, heritability estimates based on the animal model are typically about 24% lower than those based on other methods such as parent-offspring resemblance [58], so our estimate of heritability of tarsus length in house wrens (0.11) is relatively conservative. Alternatively, low heritability of tarsus may be due to high residual variance rather than low additive genetic variation [59]. A long-term study of Savannah sparrows (*Passerculus sandwichensis*), for example, has shown that heritability can vary with the age at which traits are measured, and residual variation was generally much higher in traits expressed early in development (e.g., tarsus length in nestlings) [59].

Consistent with a number of other studies employing the animal model [6,11,29,30], condition was significantly heritable, with additive genetic variation accounting for about 14% of the phenotypic variation in this trait. This result might be considered surprising, given that body condition often is subject to strong directional selection [2,6,60] and that nestling condition in our house wren population is positively correlated with recruitment to the breeding population and subsequent reproductive success [27]. However, assuming that condition is influenced by the expression of many loci, including those involved in the acquisition and efficient use of resources, this alone might sustain genetic variation in the face of strong directional selection [1]. In addition, selection may be acting primarily on the environmental component of variation in condition [6]. This occurs if the positive correlation found between condition and fitness reflects a shared dependence on environmental factors rather than a causal relationship [61].

PHA response was not significantly heritable, consistent with other studies employing the animal model to estimate additive genetic variance [11,62], as well as those relying on parent-offspring resemblance [63,64]. A prominent exception to this pattern is a study of common kestrels (*Falco tinnunculus*) employing the animal model that revealed a surprising high heritability of PHA response in fledglings (*h*^*2*^ = 0.47-0.55) [65]; as with tarsus length discussed above, such differences could be due to age-related variation in the magnitude of residual variance. Although a number of cross-fostering studies also have reported significant heritable variation in the PHA response [10,31,32], these have relied on full-sib comparisons that likely produce inflated estimates because of dominance variance and maternal effects [62].

Nest effects accounted for over half the phenotypic variation in nestling PHA response, a result consistent with previous studies in this population showing that hatching date and time of injection can influence PHA response of nestling house wrens [24,25,41,66]. Additional environmental factors might also include food availability, temperature, and parasite load [12], and we also cannot rule out the possibility of parental effects arising from differential provisioning of broods [39,55] or transgenerational priming of immunity arising from the transfer of maternal antibodies in eggs [67]. Given the complex nature of the PHA response, which involves both innate and adaptive components of the immune system [12,13], Martin et al. [12] have cautioned against interpreting larger swellings as indicative of stronger cell-mediated immunocompetence. In our house wren population, however, a multi-year study has revealed that nestlings with the strongest response to PHA injection have the highest likelihood of recruitment to the breeding population, and those with the strongest PHA responses are more likely to breed through two years of age [27]. Thus, the PHA response appears to be the focus of significant directional selection in our population and intimately associated with offspring fitness.

Nest effects had a major influence on nestling haematocrit, consistent with studies of other wild bird populations [17,20,21]. A number of environmental factors can influence nestling haematocrit, among them parasite load [19,20], food availability [16,68], and temperature [69]. Parasite load appears to be an especially important factor in other species, but ectoparasite loads in another population of house wrens did not affect nestling haematocrit [57]. In our house wren population, nestlings are frequently infested with two species of ectoparasitic mites [56] and their density varies greatly among nests [56]. Although a relationship between the density of mites and nestling haematocrit has not been established in our population, variability in the level of infestation could account for at least a portion of the nest effects detected in the present study, as it has been in other species [70]. In addition to variation in ectoparasite load, parental effects mediated through differences among broods in levels of parental provisioning of nestlings could further contribute to nest effects on nestling haematocrit [71]. Although we did not measure provisioning of nestlings in the present study, provisioning rates within and among pairs can vary widely [39,55], and thus differences among broods in the amount of food provided to nestlings could explain some of the nest effects on nestling haematocrit.

In addition to the strong environmental effect on nestling haematocrit, haematocrit was significantly heritable, in contrast to cross-fostering studies of other species [17,21] that have shown significant effects of nest-of-rearing on nestling haematocrit, but no effect of nest-of-origin. Additive genetic variation accounted for approximately 15% of the phenotypic variation in this trait. Studies that have employed cross-fostering [17,21] or parent-offspring resemblance [20] to detect genetic effects on haematocrit have relied on much smaller sample sizes than the one on which our pedigree was based, and, thus, the absence of a genetic effect in these studies may have been due to a lack of statistical power (but see [33] for evidence of a significant nest-of-origin effect in house martins). In support of this possibility, a study of a captive population of zebra finches (*Taeniopygia guttata*) based on a pedigree of similar size as the one considered here [11], reported a heritability of 0.38 ± 0.077 (*P* <0.001) as determined by the animal model.

There was a significant positive phenotypic correlation, but no genetic correlation, between nestling haematocrit and condition. This suggests that the phenotypic correlation arises because of parallel effects of the common nest environment (including parental effects) on the two traits, and not because of any underlying pleiotropic effect. The absence of genetic correlations in this study should be regarded with some caution, however, as our power to detect such correlations given our sample sizes is probably limited. This is reflected in the large standard errors associated with our *rG* estimates. Increased precision could be obtained through larger sample sizes and/or the use of cross-fostering designs that better facilitate statistical separation of additive genetic from nest effects [72]. Both nestling condition and haematocrit appear to be closely related to fitness in our population, predictive of both recruitment to the breeding population and subsequent reproductive success [27], and would thus seem to represent different axes of condition, one reflecting fat stores and the other oxygen-uptake capacity. The relationship between haematocrit and fitness is more complex than the one between condition and fitness, however, as intermediate haematocrit values result in the highest recruitment to our population, suggestive of stabilizing selection [27]. Although oxygen transport generally increases with increasing haematocrit, increases in blood viscosity beyond a certain threshold may actually hinder oxygen transport [73]. Indeed, experimental studies in mice have shown that oxygen uptake and physical endurance are maximized at intermediate values of haematocrit [73].

## Conclusion

Environmental effects played a paramount role in shaping the expression of the fitness-related traits measured in this wild population of house wrens, but two of them, condition and haematocrit, retained significant heritable variation. It is becoming increasingly evident that the maintenance of genetic variation in condition measures, in particular, appears to be a pervasive feature of wild bird populations, in apparent contradiction of conventional selection theory [6,30,74]. A major challenge in future studies will be to explain how such variation persists in the face of the directional selection acting on condition in house wrens and other species. Condition was also positively correlated with both the PHA response and haematocrit, but in the absence of any significant genetic correlations, it appears that this covariance arises through parallel effects of the environment on this suite of traits (but see [9]). This would seem to bolster the case for the utility of metrics designed to capture the multi-dimensionality of condition based on multiple physiological and morphological measures including mass, haematocrit, and immune responsiveness [3].

## Availability of supporting data

The data supporting the results of this article are available in the Dryad digital repository doi:10.5061/dryad.jk2m0 [75] (http://doi.org/doi:10.5061/dryad.jk2m0).
